# Elucidation of the binding mechanism of astragaloside IV derivative with human serum albumin and its cardiotoxicity in zebrafish embryos

**DOI:** 10.3389/fphar.2022.987882

**Published:** 2022-09-23

**Authors:** You-Jiao Wu, Zhan-Hua Li, Jiu-Yan Li, Yan Zhou, Run-Yue Wang, Xiao-Yi Chen, Lin-Sen Qing, Pei Luo

**Affiliations:** ^1^ State Key Laboratories for Quality Research in Chinese Medicines, Macau University of Science and Technology, Taipa, Macao, SAR, China; ^2^ Chengdu Institute of Biology, Chinese Academy of Sciences, Chengdu, China

**Keywords:** astragaloside IV derivative, drug-HSA interaction, equilibrium dialysis, UHPLC-MS/MS, molecular modeling, zebrafish

## Abstract

LS-102 is a new derivative of astragaloside IV (AGS IV) that has been shown to possess potentially significant cardioprotective effects. However, there are no reports concerning its interaction with human serum albumin (HSA) and toxicology in vertebrates. The present investigation was undertaken to characterize the interaction of AGS IV and LS-102 with HSA using equilibrium dialysis and UHPLC-MS/MS methods, along with computational methods. Notably, the effects of AGS IV and LS-102 were studied *in vivo* using the zebrafish embryo model. Markers related to embryonic cardiotoxicity and thrombosis were evaluated. We showed that the plasma protein binding rate of AGS IV (94.04%–97.42%) was significantly higher than that of LS-102 (66.90%–69.35%). Through site marker competitive experiments and molecular docking, we found that AGS IV and LS-102 were located at the interface of subdomains IIA and IIIA, but the site I might be the primary binding site. Molecular dynamics revealed that AGS IV showed a higher binding free energy mainly due to the stronger hydrophobic and hydrogen bonding interactions. Moreover, the secondary structure implied no obvious effect on the protein structure and conformation during the binding of LS-102. LS-102 significantly ameliorated the astramizole-induced heart rate slowing, increased SV-BA spacing, and prevented arachidonic acid-induced thrombosis in zebrafish. To our knowledge, we are the first to reveal that LS-102 binds to HSA with reversible and moderate affinity, indicating its easy diffusion from the circulatory system to the target tissue, thereby providing significant insights into its pharmacokinetic and pharmacodynamic properties when spread in the human body. Our results also provide a reference for the rational clinical application of LS-102 in the cardiovascular field.

## 1 Highlights

A comparison between astragaloside IV and LS-102 on the interaction with HSA was performed.
Equilibrium dialysis and UHPLC–MS/MS combined with *in silico* approaches was established.
Preliminary elucidation the potential mechanism of cardiovascular toxicity caused by LS-102.

## 2 Introduction

**FIGURE 1:**
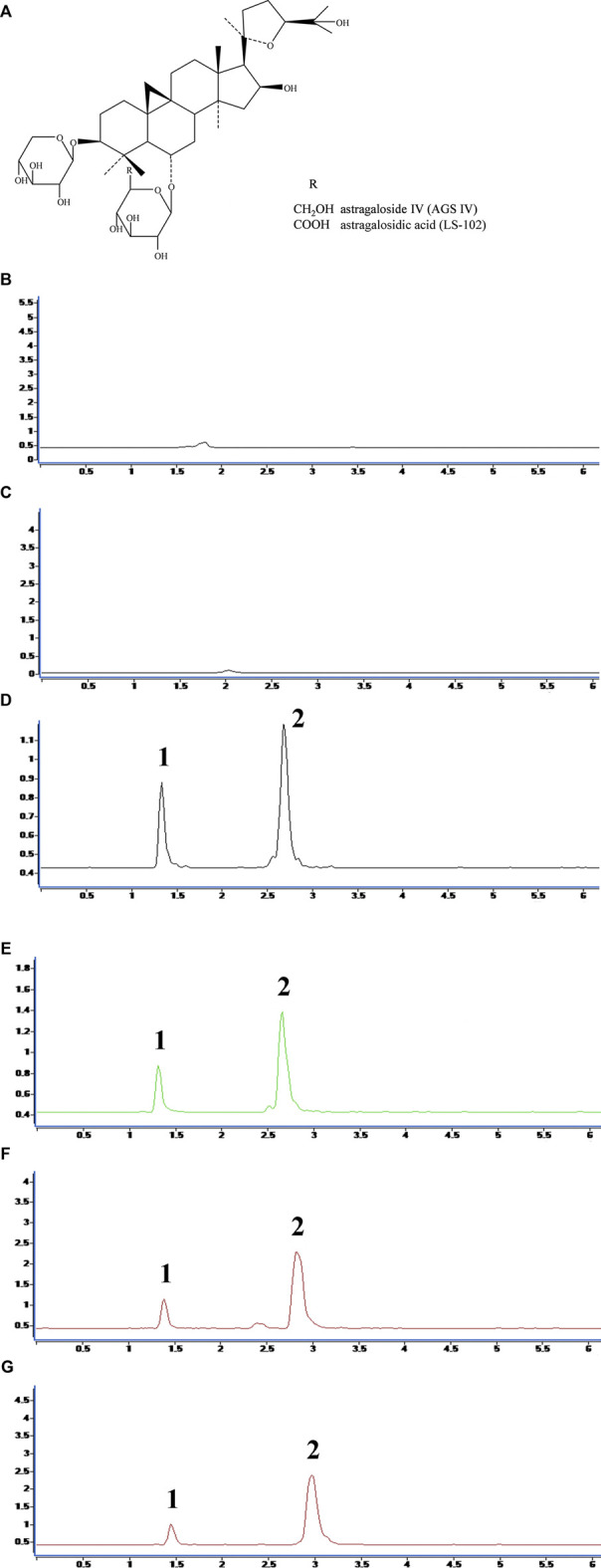
**(A)** Chemical structures of astragaloside IV (AGS IV) and astragalosidic acid (LS-102). The chromatograms for selectivity validation of LS-102. **(B)** Blank human plasma; **(C)** Blank external dialysate; **(D)** Human plasma (internal dialysate) sample from the protein binding assay; **(E)** Blank plasma spiked with LS-102 and internal standard; **(F)** External dialysate sample from the protein binding assay; **(G)** Blank external dialysate spiked with LS-102 and internal standard; 1, Digoxin; 2, LS-102.

Astragaloside IV (AGS IV, 3-*O*-β-d-xylopyranosyl-6-*O*-β-d-glucopyranosyl-cycloastragenol) is a cycloartane-type triterpenoid saponin that is considered the primary active ingredient of Astragali Radix, which is widely used in treating cardiovascular diseases in China (Ren et al., 2013). Unfortunately, AGS IV has limited efficacy, which is mainly due to its very low solubility in biological fluids, resulting in pharmacokinetic disadvantages after oral administration (Huang et al., 2006). Hence, a new water-soluble derivative of AGS IV, astragalosidic acid (LS-102), has been synthesized by our research group (Qing et al., 2017). LS-102 and AGS IV share a similar structure, except for a substitution of −COOH for −CH_2_OH on a glucose moiety (structure shown in Figure 1A). However, LS-102 demonstrated higher cardioprotective activity and bioavailability enhancement activities than AGS IV through *in vitro* and *in vivo* experiments. For instance, LS-102 exerted protective effects on cardiomyocyte damage induced by hypoxia/reoxygenation injury and alleviates myocardial ischemia reperfusion-induced injury in animal and cell models. Its cardioprotective mechanism has also been further illustrated (Chen et al., 2020). Additionally, LS-102 showed better transepithelial permeability and intestinal absorption in rodent pharmacokinetics studies, and showed no abnormalities or death in mice, even at high treatment doses (Qing et al., 2019; Sun et al., 2019). Increasing evidence indicates that LS-102 displays significant cardioprotective potential for further preclinical research. Despite the promising activities of LS-102 mentioned above, the molecular basis, cardiac function-improving effects and thromboprophylactic effects are unclear. Moreover, various other factors may influence the circulation of LS-102 in humans, including binding of the compound to plasma proteins (Lakhani, 2005).

Human serum albumin (HSA) is one of the most abundant plasma proteins available in blood and other interstitial fluids. HSA crystallographic data have revealed that it is a single polypeptide chain of 585 residues with a helical triple-domain structure. The principal function of HSA is to act as a carrier for drugs, fatty acids and metabolites to particular biological targets at its two primary binding sites (site I and site II) (Zsila et al., 2011; Wang et al., 2017). Therefore, the investigation of drug binding to HSA is crucial to understanding the pharmacodynamics and pharmacokinetic profile of a drug (Vallner, 1977). Due to the high concentration of HSA in plasma, the binding affinity of drugs to HSA is an important consideration when designing and developing new drugs (Yang et al., 2014). Additionally, the interaction of drugs that bind simultaneously to HSA can change the HSA binding behavior and potentially modulate the final therapeutic efficiency of the drugs (Tesseromatis and Alevizou, 2008). Generally, drugs with high affinity for HSA usually show a relatively slow distribution and clearance of drugs, which may extend the apparent half-life and lead to a high probability of various drug interactions (Lakhani, 2005). In contrast, the less bound a drug is, the more efficiently it can traverse cell membranes and diffuse (Benet and Hoener, 2002). To the best of our knowledge, despite the various biological effects of AGS IV, no previous study has performed an in-depth analysis of the interaction between AGS IV and HSA. Recently, XU *et al.* found that AGS IV had a very high degree of protein binding (up to 86.69%) with human plasma (Xu et al., 2016). Earlier reports demonstrated that the ratio of protein binding of drugs decreases accordingly with increasing polarity (Liang et al., 2013); thus, we raise the hypothesis that the chemical modification LS-102 will favor either the binding or release of HSA, thereby increasing its tissue distribution and ability to achieve suitable oral bioavailability in the human body. Further research on drug binding sites is needed to comprehensively understand the interaction of LS-102 with HSA, which may provide a structural basis for rationally designing drugs to use or exclude the impact of HSA on drug delivery (Petitpas et al., 2001).

Until now, various techniques have been implemented to probe the interaction of compounds on proteins *in vitro*, including fluorescence spectroscopy, (Ibrahim et al., 2010) circular dichroism spectroscopy (Yue et al., 2011), ultrafiltration, NMR (Ermakova et al., 2020), and isothermal titration calorimetry (Nevidalova et al., 2018). However, equilibrium dialysis, combined with highly sensitive assays, such as ultrahigh-performance liquid chromatography/tandem mass spectrometry (UHPLC-MS/MS), is the gold standard for investigating the direct interaction between HSA and drugs (Jiao et al., 2018). The working principle of equilibrium dialysis is that the drug can be separated from the protein solution through a semipermeable membrane. Free drug can pass through the semipermeable membrane until the dialysis reaches equilibrium, while the protein-drug complex is retained in the dialysis bag. Meanwhile, the binding rate with HSA can be calculated by the MS/MS technique to quantify the free drug concentration on both sides of the dialysis solution. This method is simple, practical, economical, and can eliminate the possible influence of nonspecific binding.

The zebrafish is a vertebrate with a high degree of genetic similarity to humans and comparable experimental results. The zebrafish model is less time consuming and less dose intensive than mammals such as rats, and the embryos are transparent and easy to observe. Additionally, zebrafish are highly reproductive, fast-growing and simple to administer. As a result, zebrafish embryos are now widely used for assessing acute and chronic toxicity, cardiovascular disease, and many other diseases. (Bournele and Beis, 2016; Raftery et al., 2017).

In this study, we aimed to evaluate the binding properties of AGS IV and LS-102 with HSA and gain deeper insight into the mechanism. The combination of equilibrium dialysis and UHPLC-MS/MS was used to determine the plasma protein binding rates, as well as to explore the binding sites of HSA in the absence and presence of probes (warfarin for site I, ibuprofen for site II). Furthermore, to verify the experimental results and better understand the binding mechanism at the molecular level, molecular docking and molecular dynamics (MD) simulations were conducted to measure the binding mode and structural basis. Additionally, the effects of AGS IV and LS-102 on cardiotoxicity and thrombosis in zebrafish embryos were compared. The zebrafish heart function was viewed by measuring the heart rate, SV-BA spacing, and cardiac cystic edema, and the effect of thrombosis prevention was examined by calculating the red blood cell staining intensity and thrombus length. To the best of our knowledge, this is the first report using these effective approaches to simultaneously explore the plasma protein binding rate and competitive effect of AGS IV or its derivative, the results of which may provide more evidence for their clinical drug development.

## 3 Materials and methods

### 3.1 Regents and materials

Astragaloside IV (AGS IV) (>98%) was isolated from Astragali Radix, and its water-soluble derivative (LS-102) (>98%) was synthesized from AGS IV in our laboratory. Digoxin (purity ≥98%, The Cat. No. D6003) purchased from Aldrich Chemical Co. (Milwaukee, United States) was utilized as an internal standard (IS). Warfarin (Cat. No. A2250), ibuprofen (Cat. No. I4883) and phosphate buffer saline (PBS) tablets (Cat. No. 524650) were purchased from Sigma. Human blood samples with K_2_EDTA anticoagulant collected from healthy volunteers were provided by the Macau Blood Transfusion Centre. An equilibrium dialysis membrane with a molecular weight cutoff of 14,000 Da was purchased from Biosharp, China. MS grade methanol and acetonitrile were purchased from Anaqua (Boston, United States). Ultrapure water was generated with a Milli-Q system (Millipore, Bedford, MA, United States). Other reagents were of the highest grade commercially available.

### 3.2 Instruments and UHPLC-MS/MS conditions

UHPLC-MS/MS was performed on an Agilent 6460 series triple quadrupole mass spectrometer (Agilent, Santa Clara, California, United States) coupled with an Agilent 1290 UHPLC system. The separation was performed on a 2.1 × 100 mm Acuity UPLC Hass C_18_ column (1.8 µm particle size) equipped with elution with a mobile phase consisting of water (A) and acetonitrile (B), with gradient elution of 36% B at 0–5 min, 36%–95% min B at 5–5.1 min, 95% B at 5.1–7 min, 95%–36% min B at 7–7.1 min, and 36% B at 7.1–11 min for separation. The total run time for the analysis was 11 min (including equilibrium). The flow rate was 0.35 ml/min, and the injection volume of the sample was set as 1 µL. The column temperature was set to 30 °C, which could afford a more stabilized peak area and retention time. The triple quadrupole mass spectrometer was operated in a positive mode with a capillary voltage of 4 kV. Nitrogen served as the collision gas, and the gas temperature was set at 400 °C. The nebulizer pressure was set at 500 V. The fragment energy of 120 V and collision energy of 35 V were set for AGS IV, LS-102, and internal standard, digoxin. The quantitation of AGS IV and LS-102 was achieved in MRM mode with *m/z* transitions of 807.5 → 495.5 for AGS IV, 821.5 → 495.5 for LS-102, and 803.5 → 387.2 for digoxin.

### 3.3 Sample preparation

Following approval by the Clinical Research Ethics Committee of Macau University of Science and Technology, the human blood samples were centrifuged at 3000 × g for 10 min at 4°C, and then the supernatants were transferred into a centrifuge tube and stored at −80 °C before the protein binding experiment. Stock solutions of AGS IV and LS-102 (both 1 mg/ml) were prepared by dissolving the compounds in methanol and stored at 4 °C until they were required for the binding rate assay. Warfarin and ibuprofen were prepared in the same manner for the competitive experiment.

### 3.4 Treatment of dialysis membranes

The dialysis membranes were processed according to the guidelines provided by the supplier. Briefly, the membranes were cut into 10 cm long sections and then boiled and soaked in deionized water for 20 min. Subsequently, the membranes were soaked in 30% ethanol for 20 min and washed completely with deionized water. Following washing, the membranes were transferred into PBS (pH 7.4) solution to activate for 60 min and finally soaked in methanol solution for storage at 4°C.

### 3.5 Plasma protein binding study

The binding rate of AGS IV/LS-102 and HSA was determined using a slightly modified equilibrium dialysis method (Metsu et al., 2020). Briefly, three concentrations of AGS IV and LS-102 were prepared separately at 0.714, 0.476, and 0.238 mg/ml for the high, medium, and low drug groups, respectively. The stock solutions of HSA (0.5 ml) and drug group (0.5 ml) were placed in a dialysis membrane bag that was ligated at both ends and then immersed in a test tube filled with 50 ml of PBS solution for 72 h at 4 °C. After equilibrium, a 0.5-ml solution of outer dialysate and internal dialysate was collected into a 2-ml EP tube, and 10 µL IS (digoxin, 0.1 mg/ml) solution was added to each tube. Both samples were added to 1.5 ml of acetonitrile and vortexed for 3 min, and then the samples were separated via centrifugation at 18,000 × g for 20 min. The supernatants were transferred to another EP tube and dried under a nitrogen stream. The residue was redissolved in 1 ml methanol, sonicated for 2 min, vortexed for 3 min, and centrifuged at 18,000 × g for 10 min. The supernatant was filtered with a 0.22 µm microporous syringe filter in a 2 ml sample vial, and 1 µL was injected into the UHPLC-MS/MS system for analysis. The plasma protein binding rate was calculated according to the following formula (Talbert et al., 2002):

$$Plasma\;binding\;rate=\frac{Internal\;drug\;concentration-External\;drug\;concentration}{Initial\;drug\;concentration\;}\times 100\%$$

### 3.6 Method validation

The method validation for the plasma protein binding rate assay included linearity, accuracy, precision, selectivity, recovery, and stability, which were validated on 3 consecutive days. The linearity of the calibration curve for the external dialysate and plasma groups was assessed. The calibration curve was generated by taking the peak area of each standard solution sample to IS as the ordinate (Y) and the mass concentration of each standard solution sample as the abscissa (X). The stock solutions of AGS IV and LS-102 were diluted with methanol into six increasing concentration groups as calibration curve standard solutions, with concentrations ranging from 0.02 to 0.17 mg/ml for plasma curves and 0.02–0.07 mg/ml for external dialysate curves. A 1 µl aliquot of sample for each group was analyzed after processing following the sample preparation procedure. The correlation coefficient, as a standard for evaluating the linearity, was considered acceptable at ≥ 0.990.

The accuracy and precision validation was evaluated via the determination of the quality control (QC) samples spiked with three concentrations of AGS IV and LS-102 (0.15 mg/ml, 0.10 mg/ml, and 0.06 mg/ml) for plasma and external dialysate. Specifically, six separately prepared QC samples at 3 concentrations were measured in 1 day for accuracy validation. One group of QC samples at three concentrations was measured six times for precision validation. The selectivity validation was evaluated by comparing the chromatograms of the blank human plasma, the blank human plasma spiked with AGS IV and the human plasma sample obtained from the plasma protein binding assay. The stability was assessed via determination of the relative standard deviation (RSD) value of QC samples spiked with three concentrations (0.15 mg/ml, 0.10 mg/ml, and 0.06 mg/ml) of AGS IV and LS-102 at different times (0, 2, 4, 8, and 12 h) stored at 4°C. Extraction recovery was determined by determining the peak area ratio of the plasma sample spiked with AGS IV and IS before the sample preparation procedure.

### 3.7 Data analysis

All chromatograms were evaluated with Mass Hunter version 1.1 software using the internal standard method and all peak area ratios of the analyte over internal standard for calculation.

### 3.8 Site marker competitive experiments

The site marker competitive experiments were performed using two classical site probes, warfarin and ibuprofen, which were selected as the markers of subdomain IIA (site I) and subdomain IIIA (site II) separately of HAS (Ghuman et al., 2005). The procedure was consistent with the method described in section *2.5*, except that the concentrations of AGS IV and LS-102 were both fixed at 0.476 mg/ml, while the external dialysis solution was changed to contain the probe with a final concentration of 0, 0.06, 0.3, 1.2 mg/ml PBS solution. Briefly, the AGS IV/LS-102 HSA mixture (0.5 ml drug and 0.5 ml HSA) was separately incubated with three different concentrations of probe to evaluate the effects of warfarin or ibuprofen on drug-protein binding influence. Three replicates of each concentration were prepared. After equilibrium dialysis, the internal and external dialysates were analyzed by UHPLC-MS/MS.

### 3.9 Molecular modeling

Molecular docking and MD simulations were applied to investigate the binding mode and stability between AGS IV/LS-102 and HSA. Docking calculations were performed using AutoDock Vina software (version 1.12) with the Lamarckian genetic algorithm (Morris et al., 1998; Xu et al., 2017). The 3D structures of AGS IV and LS-102 were optimized for energy minimization, and the crystal structure of HSA (PDB ID: 1H9Z) was obtained from the PDB BANK (http://www.rcsb.org/pdb). The ligands and water were removed from the protein using PyMOL software. Polar hydrogen atoms were added, and Geisteger charges were assigned to the receptor protein by means of AutoDock Tools version 1.5.6. A grid box was prepared for HSA to cover the binding site with a grid box size of 78 × 40 × 82 Å and a spacing of 1 Å centered at coordinates x, y, and z (25.033, 9.569, and 20.112, respectively). The saved file in the PDBQT format was used as an input in AutoDock Vina. To increase the docking accuracy, the value of exhaustiveness was set to 120, and the final number of conformations generated was set as 20. Differences between the accessible surface areas (ΔASA) of HSA before and after binding with AGS IV/LS-102 were calculated using the online server http://cib.cf.ocha.ac.jp/bitool/ASA (Warsi et al., 2021). The conformation with the lowest energy was used for post-docking analysis and subsequent MD simulations.

The MD simulations were conducted under the periodic boundary condition (PBC) using the GROMOS G54A7FF force field of the GROMACS 5.1.3 package (van Der Spoel et al., 2005; Kabiri et al., 2012). The topology parameters of AGS IV and LS-102 were built using the Automated Topology Builder (ATB) web server (Kim et al., 2016). The complex was immersed in a cubic box of extended simple point charge water molecules. The distance between the complexes and the edge of the box was set to 10 Å. Counterions (Na^+^) were added to neutralize the system. Energy minimization was performed using the steepest descent method of 10,000 steps followed by the conjugate gradient method for 10,000 steps to release conflicting contacts. The position-restrained dynamics simulation (equilibration phase) (NVT and NPT) of the system was performed at 300 K for 100 ps followed by an MD production run for 50 ns, while the pressure was maintained at 1 bar using the Parinello-Rahman coupling algorithm. The short-range Columbic and Lennard–Jones interaction energies between compound 1 and the surroundings were monitored during the course of the productive simulation step. The visualization of protein–ligand complexes and MD trajectory analysis were conducted using VMD software. The MD trajectories were saved every 2000 ps. The stability and conformational changes in the complexes were assessed through the analysis of root-mean-square deviation (RMSD), root mean square fluctuations (RMSF), radius of gyration (Rg) and secondary structure calculations. The molecular mechanics Poisson Boltzmann surface area (MM/PBSA) analysis was performed using the g_mmpbsa tool of GROMACS and was used to calculate the complex binding energies and a more precise prediction of binding modes from MD trajectories. All graphs were obtained using the Origin 85 software.

### 3.10 Animal embryos

The wild-type AB strain of zebrafish was bred by natural pair breeding, and embryos were collected 2 h and 3 days post fertilization (3 dpf), while zebrafish of the translucent Albino strain, which were mutant for the melanin allele, were bred by natural pair breeding, and embryos were collected 3 dpf. Embryos were maintained at 28°C in fish water (water quality: 200 mg of instant sea salt per 1 L of reverse osmosis water, conductivity 480–510 μS/cm; pH 6.9–7.2 and hardness 53.7–71.6 mg/L CaCO_3_). The zebrafish facility at Hunter Biotechnology, Inc. is accredited by the Association for Assessment and Accreditation of Laboratory Animal Care (AAALAC) International. This study was approved by the Animal Ethics Committee of the State Key Laboratories for Quality Research in Chinese Medicines, Macau University of Science and Technology.

### 3.11 Maximum tolerated concentration of LS-102

Zebrafish embryos at 3 dpf were randomly selected and treated with LS-102 at concentrations of 1, 10, 100, 250 and 500 μg/ml in water, while the control group was left untreated. The mortality of zebrafish was recorded for each experimental group after 24 h of treatment.

### 3.12 Evaluation of the effects of LS-102 and AGS IV on the cardiotoxicity of astemizole-induced in zebrafish embryos

#### 3.12.1 Evaluation of the effect of LS-102 and AGS IV on zebrafish morphology

A total of 270 wild-type AB zebrafish at 3 dpf were randomly selected, and 30 zebrafish were plated in each well of a six-well plates. The concentrations of LS-102 were 27.8, 83.3 and 250 µg/ml, AGS IV was 250 µg/mL. A normal control group (fish culture water) and a 4 µM astemizole model control group were also established. Ten zebrafish were randomly selected from each group, and the heart rate and venous sinus-to-arterial bulb distance (SV-BA) of zebrafish were observed under a dissecting microscope after 2 h, 6 h and 24 h of LS-102 and AGS IV treatment. Images were collected by photographing, and the ameliorative effect of LS-102 and AGS IV on astemizole-induced cardiotoxicity in zebrafish was evaluated by the statistical significance of the heart rate and the SV-BA interval index.

#### 3.12.2 Evaluation of the effects of LS-102 and AGS IV on the development of zebrafish hearts

A total of 270 wild-type AB zebrafish at 2 h post-fertilization (2 hpf) were randomly selected, and 30 zebrafish were plated in each well of a six-well plates. The concentrations of LS-102 were 27.8, 83.3 and 250 µg/ml, while that of AGS IV was 250 µg/mL. A normal control group (fish culture water) and a 4 µM astemizole model control group were also established. After LS-102 and AGS IV were cotreated with astemizole for 48 h, zebrafish heart development was observed and photographed under a microscope to evaluate the ameliorative effect of LS-102 and AGS IV on astemizole-induced toxicity in zebrafish heart development.

### 3.13 Evaluation of the effects of LS-102 and AGS IV on arachidonic acid-induced thrombosis in zebrafish

A total of 270 melanin allele mutant translucent albino strain zebrafish at 3 dpf were randomly selected, with 30 zebrafish per well in six-well plates. The concentrations of LS-102 were 6.25, 12.5, 25, 50, and 100 µg/ml, while that of AGS IV was 100 µg/ml, and the positive control drug aspirin was 25 µg/ml. After treatment with LS-102 and AGS IV for 3 h, arachidonic acid aqueous solution was administered for 1.5 h to induce a thrombosis model in zebrafish in all experimental groups except the normal group. After staining with *o*-anisidine, zebrafish were randomly selected from each experimental group and observed under a dissecting microscope and photographed. Data were collected to qualitatively evaluate thrombosis in zebrafish, and the intensity of cardiac erythrocyte staining (length of venous thrombus *vs.* number of cardiac erythrocytes) in zebrafish was analyzed and counted to establish, the preventive effect of LS-102 and AGS IV with aspirin on thrombosis in zebrafish. The formula was calculated as follows:

Thromboprophylactic effect (%) =

$$\frac{S\;\left(drug\;administration\;group\right)-S\;\left(model\;group\right)}{S\;\left(nomal\;control\;group\right)-S\;\left(model\;group\right)}\times 100\%$$

#### 3.14 Statistical analysis

For zebrafish experiments, statistical analyses were performed in triplicate, and statistical treatments were expressed as mean ± standard deviation (SD). All statistical analyses were performed using GraphPad Prism 9.3.1 statistical software for ANOVA and Dunnett’s *t*-test to determine significant differences (*p* < 0.05).

## 4 Result

### 4.1 Method validation

#### 4.1.1 Optimization of equilibrium dialysis conditions

A temperature of 37°C and 4°C is usually necessary to achieve a steady state or equilibrium between bound and free concentrations in the equilibrium dialysis test (Metsu et al., 2020). However, the poor reproducibility of the test results when equilibrated at 37°C was experimentally investigated in this study, while the sample reached equilibrium after incubation for 72 h at 4°C with a relatively stable result. Therefore, a temperature of 4°C and a dialysis time of 72 h were used for all subsequent equilibrium dialysis experiments.

In the plasma protein binding rate study, plasma protein leakage into the external dialysate could occur due to improper operation of the experiment or other reasons, which will affect the determination of the research results. Therefore, the solution needs to be tested for leaks before sample processing. The turbidimetric method was used to determine whether protein leakage was present (Ruiter et al., 2012). After equilibrium, 1 ml of the external dialysate was collected in a transparent test tube, and then 0.5 ml of 20% trichloroacetic aid was added and observed in a dark background. If a white flocculent precipitate appeared, then protein leakage was considered to have occurred.

#### 4.1.2 Linearity and selectivity

The regression equation and correlation coefficient for the internal and external dialysates of the AGS IV and LS-102 calibration curves are shown in Supplementary Table S1. All correlation coefficient values were >0.999, which indicates decent linearity for the calibration curves in a certain range. The selectivity validation was evaluated by comparing the chromatograms, which showed that there were no endogenous interfering substances in the matrix by comparing Figure 1B and Figure 1C. Furthermore, no exogenous interference was inspected in the result after analyzing the chromatograms of Figure 1D and Figure 1E. Similarly, no interference from external dialysate could be observed later in scrutinizing Figure 1F and Figure 1G.

#### 4.1.3 Accuracy, precision, and extraction recovery

The precision, accuracy, and extraction recovery values of three QC samples with different concentrations of AGS IV and LS-102 are listed in Supplementary Table S2. The precision was measured by RSD, and the accuracy was expressed as the relative error (RE) (Lu et al., 2019). Supplementary Table S2 shows that the precision values range from 3.23% to 11.56%, and all accuracy values were within ±13.22%, suggesting that all precision and accuracy values are acceptable. The extraction recovery rate of AGS IV and LS-102 remained in the range of 85%–117%, and there was no orthogonal relationship between the recovery rate and concentration of each QC sample.

### 4.2 Plasma protein binding rate

The AGS IV/LS-102 HSA binding properties were studied by the equilibrium dialysis method. During incubation, components of the drug group could freely interact with HSA inside the dialysis bag, while the excess drugs could freely diffuse to the outside. Therefore, after equilibrium dialysis, the difference between the amounts of the external and internal drugs in the dialysis bag was detected to obtain the plasma binding rate (Zhang et al., 2012). As shown in Table 1, Student’s *t* test proved no significant difference within the concentration groups of human plasma (*p* > 0.05). The binding rates were 94.62% ± 2.70%, 97.42% ± 1.91%, and 94.06% ± 1.02% for AGS IV and 68.24% ± 1.01%, 69.35% ± 0.97%, and 68.57% ± 1.19% for LS-102. The results showed that there was no drug concentration dependence between the binding process of both AGS IV and LS-102 with HSA, which provides an experimental basis for clinical medication.

**TABLE 1:** The protein binding rates of AGS IV and LS-102 at different concentrations (n = 3).

Concentration (mg/ml)	Protein binding rate (%, Mean±SD)	
	AGS IV	LS-102
0.238	94.62 ± 2.70	68.24 ± 1.01^**^
0.476	97.42 ± 1.91	69.35 ± 0.97^**^
0.714	94.06 ± 1.02	68.57 ± 1.19^**^

^**^ *p* < 0.01, vs. analytes AGS IV.

Generally, albumin binding events play a vital role in the transport and distribution of drugs; only free drugs can act on target organs through biomembranes and are responsible for pharmacological activities, while bound drugs have no pharmacological effects. Theoretically, drugs with a high plasma protein binding rate require correspondingly higher doses to achieve an effective concentration *in vivo*, which are prone to safety problems. In other words, when combined with other strongly HSA-binding drugs, the exponentially increasing free drug concentration caused by changes in protein binding may have side effects or toxicity (Lombardo et al., 2002). Accordingly, the binding rate of AGS IV in HSA is always higher than 94% at the three concentrations, indicating that AGS IV could be easily stored and transported in the circulatory system by HSA, which is consistent with previous reports (Xu et al., 2016). In contrast, LS-102 binds moderately to HSA in the range of 68.24%–69.35%, which is significantly lower than that of AGS IV (*p* < 0.01), implying that an appropriate amount of unbound LS-102 remains in the blood circulatory system. Hence, the dissociative LS-102 can reach the target tissue to achieve the pharmacological effects of the drug.

In short, attention should be given to the variation in the free drug concentration caused by the drug combination or endogenous ligand binding. Detailed molecular information on HSA binding sites may help to evaluate the cooperative effects given that HSA has a limited number of high-affinity binding sites (Yang et al., 2014). However, as the binding sites of AGS IV and LS-102 on HSA were unclear, competitive displacement experiments were subsequently performed.

### 4.3 Competitive binding experiments using the equilibrium dialysis method coupled with UHPLC-MS/MS

Generally, HSA has two principal ligand-binding sites located in the hydrophobic cavity, namely, site I (termed the warfarin site) and site II (termed the ibuprofen site). In particular, triterpenoids such as saikosaponin C, glycyrrhetinic acid and oleanolic acid have been proven to bind to site I by fluorescence, site marker competitive or molecular docking research (Tang et al., 2006; Chen et al., 2016; Abboud et al., 2017). In this study, a simple and efficient modified equilibrium dialysis method coupled with UHPLC-MS/MS (Liu et al., 2013; Yang et al., 2014; Yu et al., 2016) was first used to identify the AGS IV and LS-102 binding sites on HSA. By monitoring the changes in the AGS IV/LS-102 HSA binding rate in the absence and presence of probes and then calculating the percentage of probe displacement, the drug binding site can be predicted.

The competitive binding experimental results are shown in Table 2. Concentration-dependent binding was not observed over the selected concentration range. Following the addition of different levels of warfarin into the HSA–LS-102 solution, the binding rates were significantly reduced compared to those without warfarin *(p* < 0.01). This indicates that the addition of warfarin significantly affected the binding of LS-102 to HSA, thereby increasing the free concentration of LS-102 and enhancing its effect, which suggests that LS-102 and warfarin share the same binding site (site I). The binding site of LS-102 is thought to be subdomain IIA of HSA (He et al., 2008). In contrast, in the presence of ibuprofen, the binding rate of the HSA–LS-102 system increased slightly at doses of 0.3 and 1.2 mg/ml, which indicates that the introduction of ibuprofen into HSA–LS-102 cannot compete with LS-102 but conversely promotes LS-102 and HSA. This is similar to previous studies that have shown that ibuprofen binding to HSA provokes the enhanced binding of (*S*)-lorazepam acetate (Fitos et al., 1999). Moreover, in the AGS IV binding system, the combination of warfarin affects the HSA–AGS IV interaction and consistently reduces the binding rates. Interestingly, the binding rates changed when ibuprofen was administered, with a slight decrease in the binding rates at concentrations of 0.06 and 0.3 mg/ml and an increase at 1.2 mg/ml, suggesting that AGS IV mainly attaches to both sites I and II of HSA.

**TABLE 2:** Plasma protein binding of AGS IV and LS-102 plus site markers in HSA (‾ x ± s, n = 3).

Analyte	Without site markers	With warfarin (mg/ml)			With ibuprofen (mg/ml)		
		0.06	0.3	1.2	0.06	0.3	1.2
AGS IV	96.67±1.14	94.90±1.95	91.10±1.06^*^	92.19±2.38^*^	92.82±1.44	93.87±2.26	97.19±1.37
LS-102	66.90±0.93	53.29±2.14^**^	58.93±0.98^**^	54.70±1.25^**^	63.22±0.72	75.16±1.99^*^	72.03±1.83^*^

^*^ *p* < 0.05.

^**^ *p* < 0.01, vs. analytes without site markers.

Sites I and II are similar in size and shape, possessing elongated hydrophobic pockets with cationic amino acid residues near their entrances. Generally, site I preferentially integrates with bulky heterocyclic ligands, and site II preferentially recognizes aromatic compounds. However, these structural features are not strict prerequisites for site binding since many drugs bind to both binding sites, albeit with different binding energies (Petitpas et al., 2003). Based on site marker experiments, we found that AGS IV and LS-102 are likely to attach to both sites I and II of HSA, but that site I may be the primary binding site, especially the binding rates of HSA–LS-102, which showed significant displacement by warfarin, whereas ibuprofen did not.

### 4.4 Molecular docking study

Next, molecular docking was applied to simulate AGS IV/LS-102 and HSA to obtain more information about the binding interaction between ligands on HSA at the atomic level and validate the experimental results (Trott and Olson, 2010). The structure of HSA has three homologous domains, and domain I is composed of residues 1–195, II (196–383), and III (384–585). Each domain is further divided into two subdomains (named A and B) to comprise six subdomains, IA, IB, IIA, IIB, IIIA, and IIIB. The two subdomains in each domain constitute a hydrophobic cavity. HSA contains two principal drug binding sites, sites I and II, which are located in the specialized cavities of subdomains IIA and IIIA, respectively. The sole tryptophan residue (Trp 214) of HSA is in subdomain IIA, which contains a large hydrophobic cavity that many drugs can bind to (Xiang et al., 2016).

**FIGURE 2:**
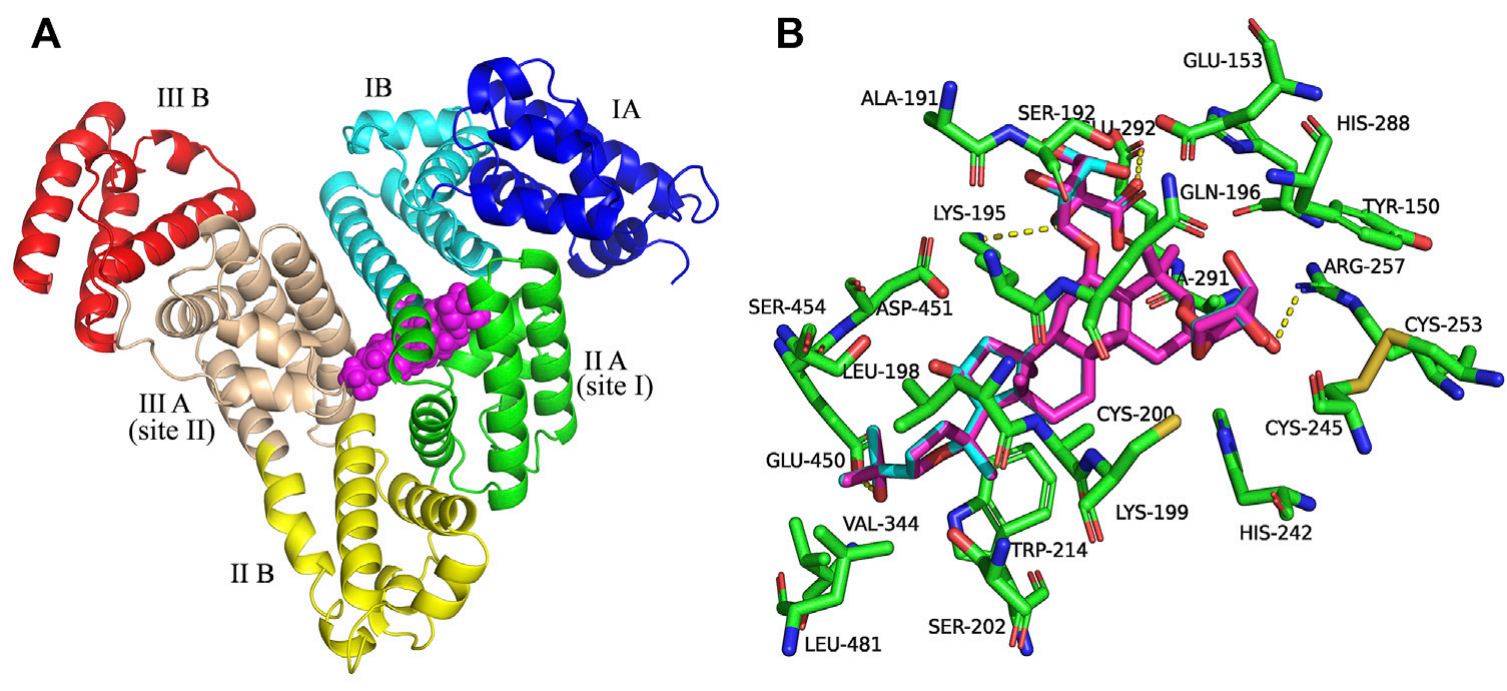
**(A)** Superposition of the structures of AGS IV and LS-102 (magenta spheres) within the active site pocket of human plasma albumin (HSA) (PDB ID: 1H9Z). Cartoon representation of HSA showing the six subdomains remarked with the following colors: IA, blue; IB, cyans; IIA, green; IIB, yellow; IIIA, wheat; IIIB, red. **(B)** The binding site was enlarged to show the superposition structures of AGS IV and LS102, with major amino acid residues (green sticks) surrounding ligands within 4 Å. The cyan structure stands for AGS IV and magentas is for LS-102, the yellow dotted lines indicating hydrogen bonds.

Figure 2 shows the best docking models of AGS IV and LS-102 for HSA. The AGS IV and LS-102 molecules superimposed well and were located at the interface of subdomains IIA and IIIA in the center of the serum albumin molecule, with the structures primarily buried in the subdomain IIA hydrophobic cavity. We also found that subdomain IIIA is an open pocket in which part of the hydroxyl group on the five-membered ring of ligands can fit into (Figures 2A,B). Hydrophobic and hydrogen were shown to be the most significant interactions of the binding mode, with approximately 23 residues located within a 4 Å distance from the ligands (Figure 2B). The cycloastragenol fused ring of these tetracyclic triterpenoids was inserted into the hydrophobic cavity of the site pocket formed by Lys-199, Trp-214, Val-344, Glu-450, Leu-198, Asp-451, Lys-195 and Ala-291, while the xylopyranosyloxy and glucopyranoside were situated in a larger hydrophobic cavity formed by Cys-245, Cys-253, His-288, Glu-292, Ser-192 and Gln-196 (Chen et al., 2014). Additionally, several hydroxy groups of AGS IV generate a hydrogen bond network with the polar residues present in the site pocket, including Lys-195, Arg-257 and Glu-292, with bond distances of 3.3 Å, 3.1 Å and 2.8 Å, respectively. Furthermore, a carboxyl and hydroxyl group of LS-102 formed H-bonds with two residues present at the inner site I pocket, including Lys-195 and Arg-257, with bond distances of 3.3 Å and 3.2 Å, respectively.

Therefore, the moderate interaction of hydrophobic and hydrogen bonds in the active site pockets is considered essential for the stable conformation of the HSA-ligand complex, while the binding sites of subdomains IIA and IIIA are preferentially involved. This is in agreement with the result of a previous study showing that hydrophobicity and hydrogen bonds are significant factors that control triterpenoid-albumin binding (Maciazek-Jurczyk et al., 2018). Overall, the above analysis fits extremely well with the site experimental results. Thus, our study offers a rational molecular explanation for the site competitive binding experiment in which increasing warfarin and ibuprofen concentrations in the drug group-HSA system influenced the binding rates.

The ASA values of residues were calculated for HSA, HSA-AGS IV, and HSA-LS-102 and are presented in Supplementary Table S3. It was found that the average values of ΔASA for the residues near the binding sites were approximately 42.02 Å^2^ and 42.17 Å^2^ for AGS IV and LS-102 in the binding interaction, respectively, both of which are much greater than 10 Å^2^, indicating that AGS IV and LS-10 effectively and tightly bind to the active sites of HSA (Wang et al., 2011). The residues Lys-195, Arg-257 and Glu-292 of HSA suffer a considerable change (90.48%, 64.83%, and 47.02%, respectively) in ASA due to the hydrogen bonding interactions with LS-102 as might be expected. The loss of ASA by hydrophobic residues is 14.05%, 43.27%, 43.31%, 62.26%, 76.71%, 90.82%, 91.57%, and 95.24% for Glu-450, Gln-196, His-288, Ser-192, Ala-291, Trp-214, Asp-451, Lys-199 and Leu-198, respectively. Therefore, ASA calculations may prove that hydrogen bonds and hydrophobic interactions play essential roles in the binding of AGS IV/LS-102 to HSA, which is in agreement with the results of the docking study.

Notably, there is a significant difference between AGS IV and LS-102 when binding with HSA according to the plasma protein binding experiments, while the docking results here show that AGS IV and LS-102 overlap very well and are located in the same pocket of HSA. We suspect that the carboxyl group of LS-102 improves the metabolic stability and through allosteric modulation arise among the binding sites.

### 4.5 Analysis of the dynamic trajectories

**FIGURE 3:**
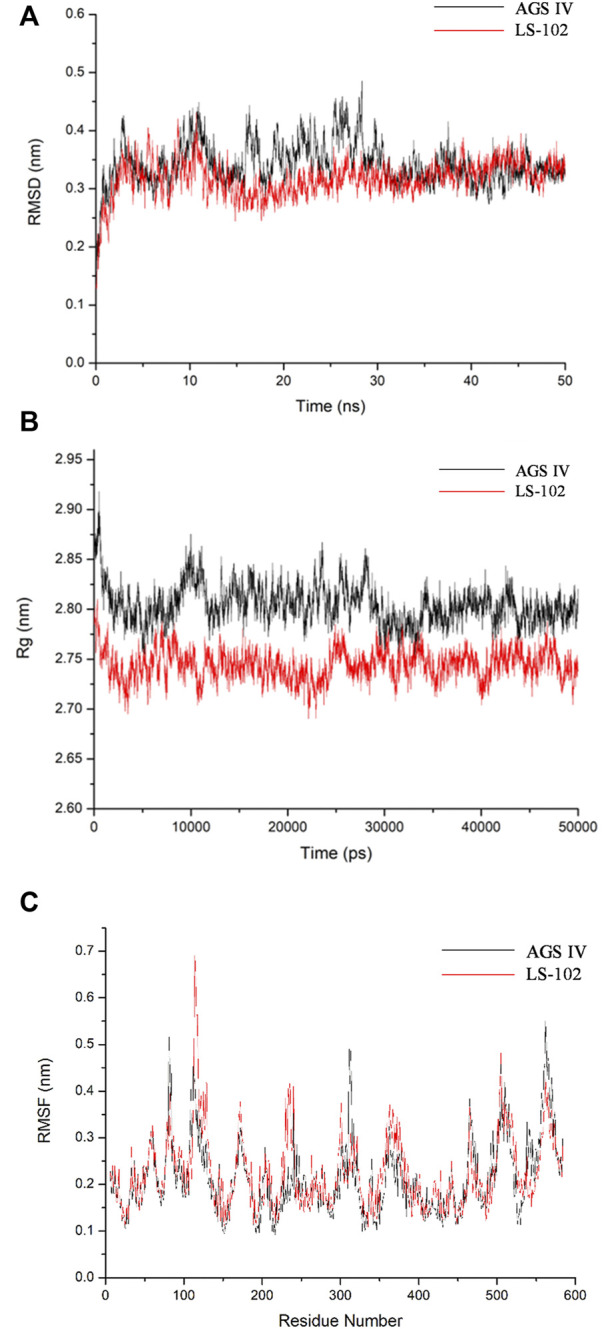
Molecular dynamics simulation of HSA–ligand complex for 50 ns time scale **(A)** Root-mean-square deviation (RMSD) of Cα-atoms for HSA-AGS IV/LS102 complex. **(B)** Radius of gyration values for HSA present in systems. **(C)** RMSF values of HSA/AGS IV and HSA/LS-102 complex were plotted against residue numbers.

Molecular docking is a static mode of ligand binding; hence, coupling docking with MD simulation can further elucidate the dynamic behavior and possible geometric changes in the AGS IV/LS-102 HSA complex under simulated physiological conditions. Through the analysis of the output trajectories, dynamic structural properties such as the RMSD, Rg and secondary structure were obtained to evaluate the stability and conformational changes of the HSA-ligand complex (Lakshmi et al., 2017). The RMSD was calculated to understand the protein stability by superimposing C-alpha atoms in the active site pocket along the 50 ns MD trajectories (Figure 3A). For all systems, the RMSD values varied between 2.5–5 Å, and both reached stability in the first 12 ns time scale. The fluctuations were relatively small during the remainder of the simulation process, with a slight fluctuation between 15 and 30 ns in the HSA–AGS IV complex. The results demonstrate that the conformation of HSA in the AGS IV/LS-102 complex was slightly changed, and that the simulation time is sufficient for the refinement in the period used. Additionally, the Rg value for the complex against time scale was determined and is shown in Figure 3B. Generally, Rg represents the HSA compactness, and a higher Rg value indicates a looser structure in a specific region of the protein. During the simulation, the Rg plot showed no significant change in either system, but the average Rg values of HSA in AGS IV (approximately 2.80 nm) were slightly higher than those in LS-102 (approximately 2.75 nm), revealing that the combination of LS-102 and HSA made HSA more compact than in AGS IV. The RMSF plots indicate the fluctuation of residue relative to the average position of the protein to a certain extent, and the result can be used as the data index to study the dynamic movement of the protein–ligand complex (Gan et al., 2018). RMSF analyses were performed based on the 50 ns trajectory and plotted against all residues for the HSA-AGS IV and HSA-LS-102 systems (Figure 3C). As shown, residues within the loop regions of HSA-AGS IV and HSA-LS-102 display higher flexibility, and a similar pattern of flexibility is observed for these complexes. The binding affinity of HSA molecules can be influenced by amino acid residues (Senthilkumar et al., 2016). The RMSF values for binding site I remained low, indicating that these residues are rigid compared with other residues, and this suggests that the binding affinity of site I can be affected by AGS IV/LS-102 binding.

**FIGURE 4:**
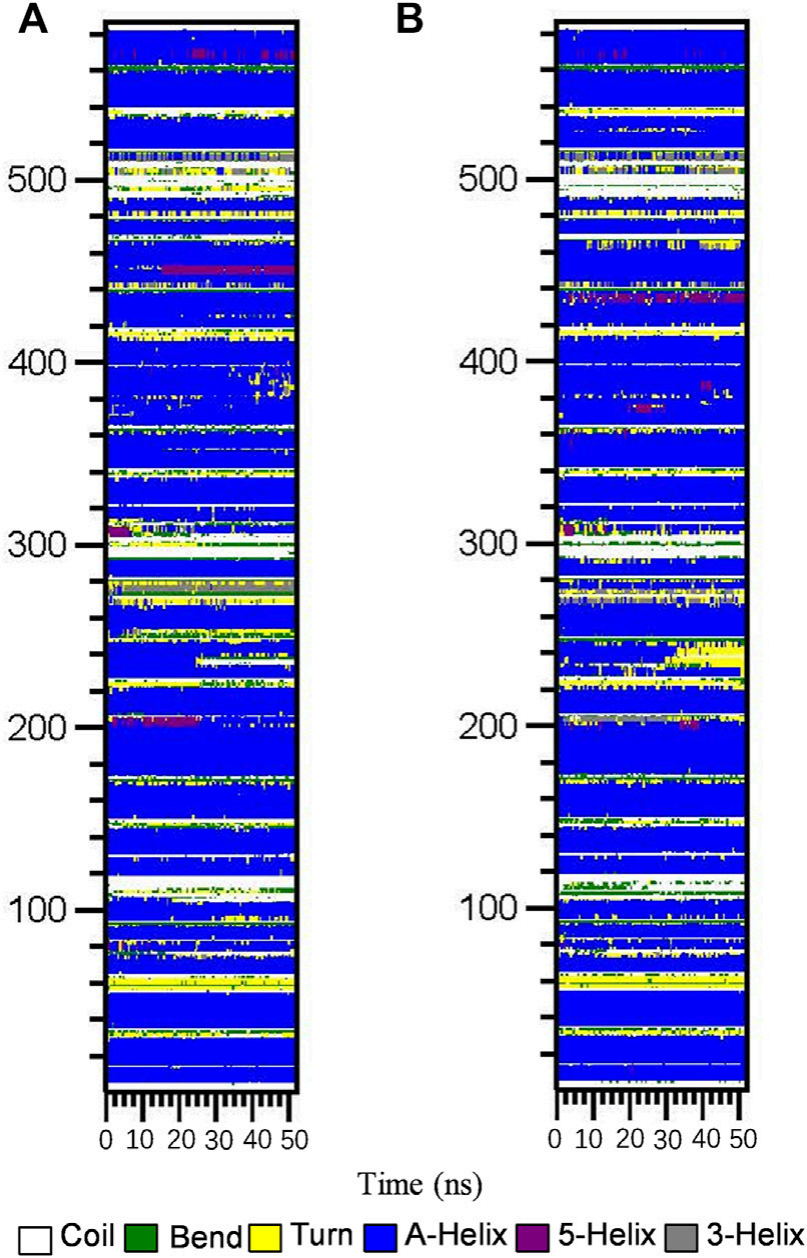
Structural changes of HSA-AGS IV **(A)** and the HSA-LS102 **(B)** complex during simulation

Subsequently, the DSSP method was used to investigate the changes in the secondary structure of proteins (Moradi et al., 2018). Figure 4 shows the structural changes of the HSA–ligand complex during simulation. Structural displacements are observed in some regions, while some of the α-helix contents changes to a 5-helix. The percentages of different structure types are listed in Supplementary Table S4 and are mainly composed of A-helix, of which AGS IV is 0.69 and LS-102 is 0.70, along with some Bend, Turn, 3-Helix and 5-Helix. This observation showed that the differences between HSA binding to AGS IV and LS-102 are negligible, which indicates that there is no substantial change in the secondary structure of the protein upon interacting with the two ligands. Moreover, although the whole secondary structure of HSA-LS-102 increased by approximately 0.01, the bend content decreased within the same range. These observations strongly emphasize the existence of a safe interaction between AGS IV and LS-102 in the HSA complex, whereas LS-102 has no adverse effect on protein structures and conformation.

Next, MM/PBSA analysis was performed to calculate the main driving force for physical adsorption of the complex (Yu et al., 2016), the results of which are listed in Table 3. Obviously, van der Waals, electrostatic, polar solvation and solvent accessible surface area (SASA) energy make the greatest contributions to the total binding-free energy. The results showed that the HSA/AGS IV complex provides more negative van der Waals energies (−340.247 kcal/mol) than the LS-102 complexes (−321.458 kcal/mol), which may be due to the stronger hydrophobic interactions. Therefore, we conclude that van der Waals forces are the driving force for binding. Although electrostatic energy (−72.705 and −116.843 kJ/mol for AGS IV and LS-102, respectively) is also favorable forbinding in a vacuum, it is offset by the polar solvation energy (313.271 and 391.229 kJ/mol) when considering the solvation effect. A positive polar solvation energy shows an unfavorable electrostatic effect on solvation; this may be the result of the hydrophobic interactions of the ligand in HSA, which also resulted in negative SASA energy (−36.313 and −35.977 kcal/mol for AGS IV and LS-102, respectively). Finally, the binding conformations of AGS IV exhibited lower binding energies (−135.994 kcal/mol) compared to LS-102 (−83.049 kcal/mol), indicating that AGS IV had a stronger binding ability of HSA than LS-102, which is in balance with the results of the plasma protein binding studies.

**TABLE 3:** Calculated values that contributed to binding-free energy from Poisson Boltzmann surface area (MM/PBSA).

Analytes	Van der waal energy	Electrostatic energy	Polar solvation energy	SASA energy	Binding energy
AGS IV	−340.247±16.998	−72.705±22.211	313.271±42.201	−36.313±1.665	−135.994±26.807
LS-102	−321.458±20.459	−116.843±26.138	391.229±52.868	−35.977±1.570	−83.049±31.142

All values, standard deviations and averages, are expressed in kcal mol^−1^

### 4.6 Maximum-tolerated concentration (MTC) of LS-102 and AGS IV in zebrafish

Zebrafish have become widely used as biological models in drug toxicity tests (Bournele and Beis, 2016). As shown in Supplementary Table S5, the MTC assay showed no mortality in zebrafish at concentrations of LS-102 ranging from 1 to 100 µg/ml, and 13.3% and 100% mortality at concentrations of 250 and 500 µg/ml, respectively. Therefore, the MTC of LS-102 used in the experiment was determined to be 100 μg/ml.

### 4.7 Comparison of LS-102 and AGS IV on astemizole-induced cardiotoxicity in zebrafish embryos

Zebrafish have become immensely powerful model organisms for uncovering mechanisms of cardiac development and function (Liu and Stainier, 2012). In the current study, we used astemizole to induce cardiotoxicity in zebrafish embryos to detect the corresponding cardiac performance alterations. After treatment of LS-102 and AGS IV with 4 μM of astemizole, the heart rate and SV-BA spacing of zebrafish in the model group were compared with those of the normal group, *p* < 0.001, indicating successful modeling. After treatment of LS-102 and AGS IV with 4 µM of astemizole for 2 h and 6 h, respectively, LS-102 improved the slowed heart rate and increased the SV-BA spacing in zebrafish at 27.8, 83.3, and 250 µg/ml concentrations. After 24 h, LS-102 improved the slowed heart rate at 250 µg/ml and the increase in SV-BA spacing at 83.3 and 250 µg/ml, while the AGS IV group showed no significant changes.

**FIGURE 5:**
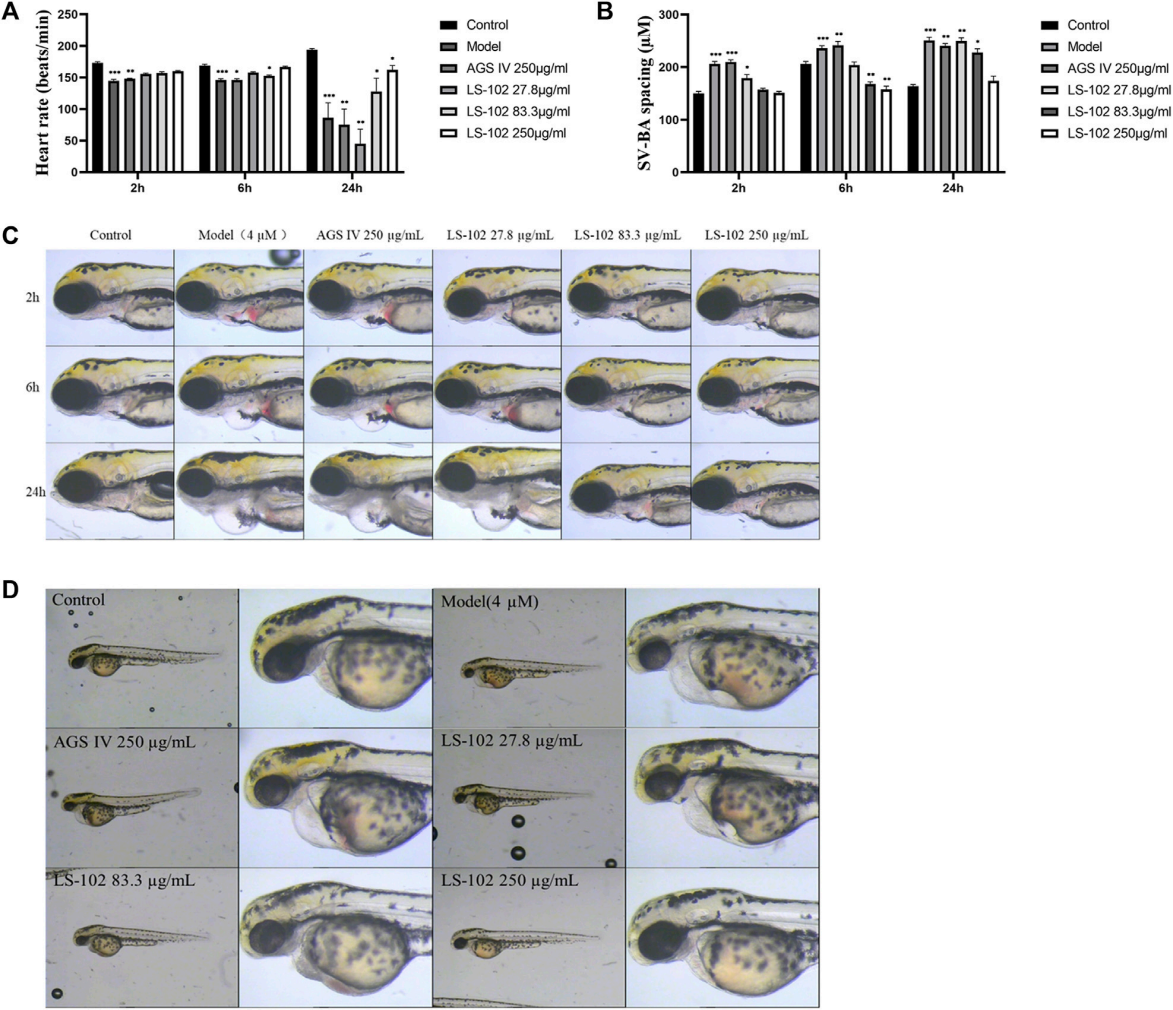
**(A)** Changes in heart rate of zebrafish after induction of 4 µM astemizole by LS-102 and AGS IV, respectively. **(B)** Zebrafish SV-SA spacing after cotreatment of LS-102 and AGS IV with 4 µM astemizole. **(C)** Typical picture of SV-BA spacing after cotreatment with AGS IV and LS-102 and 4 µM astemizole. **(D)** Typical picture of zebrafish heart after cotreatment of AGS IV and LS-102 with 4 µM astemizole. ^*^ *p* < 0.05, ^**^ *p* < 0.01, ^***^ *p* < 0.001, vs. Control group.

After treatment of zebrafish with LS-102 and AGS IV and 4 µM astemizole for 48 h, 100% of zebrafish in the model group developed cardiac vesicle edema and blood flow deficiency. In the 27.8 µg/ml LS-102 concentration group, 100% of zebrafish showed cardiac vesicle edema and loss of blood flow. In the 83.3 µg/ml concentration group, 83.3% showed pericardial edema and venous sinus stasis, 26.7% showed a loss of blood flow, and 56.7% showed slow blood flow. In the 250 µg/ml concentration group, the zebrafish mortality was 30%, but the zebrafish showed no significant toxicity. This indicated that 83.3 and 250 µg/ml of LS-102 had an ameliorating effect on the developmental toxicity of zebrafish hearts induced by 4 µM astemizole. The results are shown in Tables 4, 5; Supplementary Table S6 and Figure 5.

**TABLE 4:** Quantitative results of LS-102 and AGS IV on heart rate in zebrafish after astemizole induction (n = 10).

Inducer concentration (µM)	Groups	Concentrations (*µ*g/ml)	Heart rate (beats/min) (Mean±SE)		
			2 h	6 h	24 h
4	Control	−	173 ± 2	169 ± 2	194 ± 2
	Model	−	145 ± 2^***^	146 ± 2^***^	86 ± 24^***^
	AGS IV	250	148 ± 1^**^	146 ± 2^*^	75 ± 25^**^
	LS-102	27.8	156 ± 1	158 ± 1	45 ± 23^**^
		83.3	157 ± 2	153 ± 1^*^	128 ± 21^*^
		250	160 ± 1	167 ± 1	162 ± 7^*^

^*^ *p* < 0.05.

^**^ *p* < 0.01.

^***^ *p* < 0.001, vs. Control group.

**TABLE 5:** Quantitative results of LS-102 and AGS IV on SV-BA spacing after astemizole induction in zebrafish (n = 10).

Inducer concentrations (µM)	Groups	Concentrations (*µ*g/ml)	SV-BA spacing (*µ*m) (Mean±SE)		
			2 h	6 h	24 h
4	Control	−	150 ± 4	149 ± 5	164 ± 3
	Model	−	206 ± 5^***^	236 ± 5^***^	251 ± 6^***^
	AGS IV	250	210 ± 4^***^	242 ± 7^**^	241 ± 4^**^
	LS-102	27.8	179 ± 7^*^	204 ± 6	250 ± 6^**^
		83.3	157 ± 3	168 ± 4^**^	228 ± 7^*^
		250	151 ± 3	158 ± 6^**^	174 ± 9

^*^ *p* < 0.05.

^**^ *p* < 0.01.

^***^ *p* < 0.001, vs. Control group.

### 4.8 Comparison of LS-102 and AGS IV on arachidonic acid-induced thrombosis in zebrafish

**FIGURE 6:**
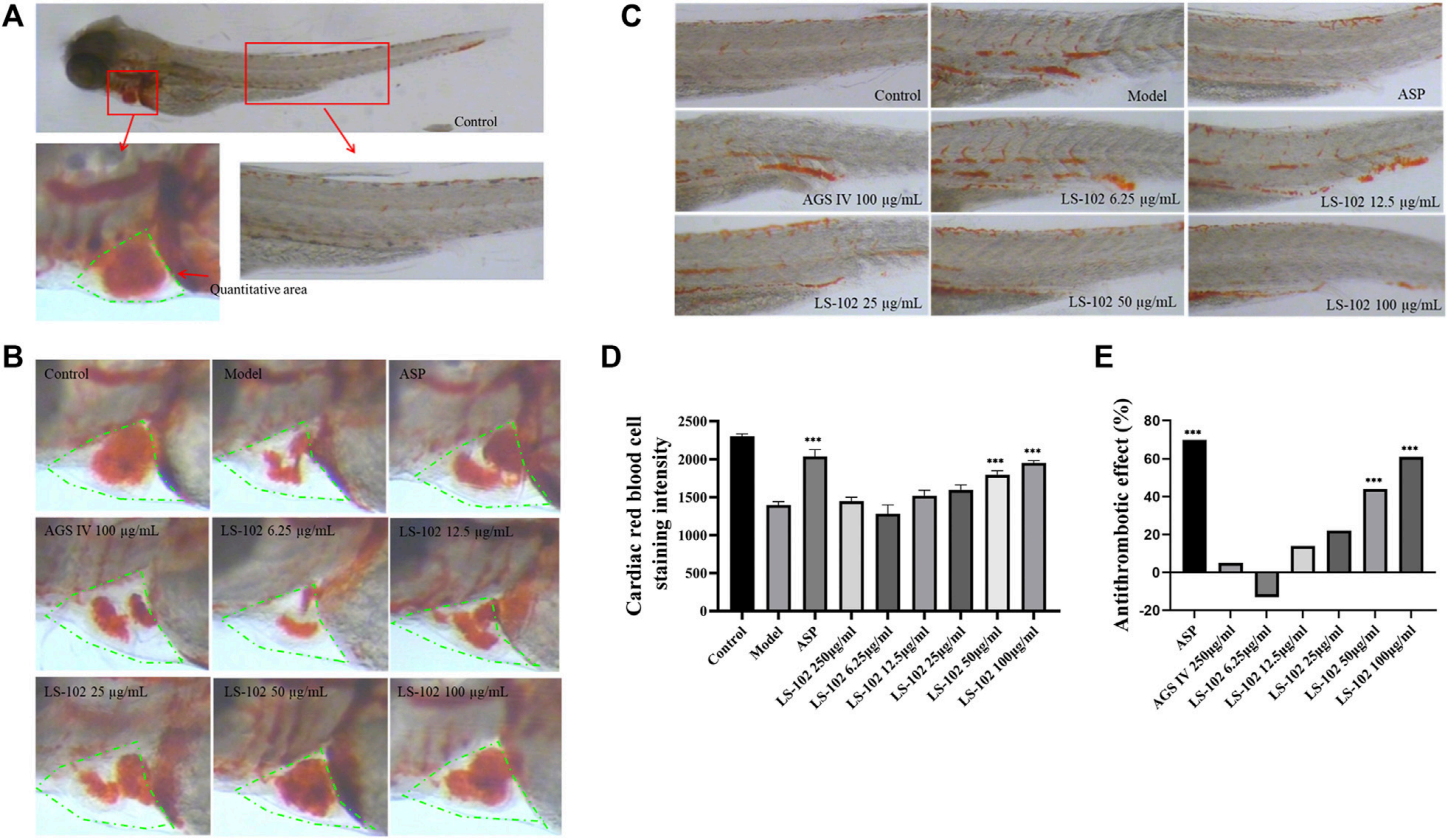
**(A)** 3 dpf Albino strain zebrafish stained with *o-Dianisidine* (green area is the quantitative red blood cell area). **(B)** Typical staining of zebrafish heart red blood cells after AGS IV and LS-102 treatment. **(C)** Typical graph for qualitative evaluation of zebrafish thrombosis after AGS IV and LS-102 treatment. **(D)** Erythrocyte staining intensity (pixels) in zebrafish hearts treated with LS-102 and AGS IV. **(E)** Thrombosis prevention in zebrafish treated with LS-102 and AGS IV. ^***^ *p* < 0.001, vs. Model group.

Zebrafish is a useful model for thrombosis studies because their platelet function shares many similar features to that in humans, including the function of arachidonic acid metabolism enzymes (Lang et al., 2010). The staining intensity of zebrafish heart erythrocytes in the model group (1397 pixels) compared to the control group (2305 pixels; *p* < 0.001), indicated successful modeling. The intensity of erythrocyte staining in zebrafish hearts of the positive control drug 25 μg/ml of the aspirin group (2036 pixels) compared to the model group (*p* < 0.001), and the preventive effect of the aspirin group on thrombosis in zebrafish was 70%, indicating the preventive effect of aspirin on thrombosis. The preventive effect of AGS IV on thrombosis was insignificant at 5% compared to the model group (*p* > 0.05). Notably, the intensity of erythrocyte staining at concentrations of 6.25, 12. 5, 25, 50, and 100 μg/ml of LS-102 was 1280, 1521, 1597, 1796 and 1950 pixels, respectively, and the preventive effect on thrombosis was −13%, 14%, 22%, 44%, and 61%, compared to the model group. (*p* > 0.05 for the 6.25, 12.5 and 25 µg/ml concentration groups and *p* < 0.001 for the 50 and 100 µg/ml concentration groups). These results indicate that LS-102 at concentrations of 50 and 100 µg/mL has a preventive effect on arachidonic acid-induced thrombosis in zebrafish. The results are shown in Table 6 and Figure 6. In summary, LS-102 was shown to ameliorate the heart rate slowing and SV-BA interval increase and prevent thrombosis in zebrafish, which may indicate its cardioprotective activity (Lu et al., 2021). As the drug-HSA binding rate is an important factor in evaluating efficacy and safety, LS-102, with moderate binding affinity to HSA may increase its tissue distribution and thus improve bioavailability, which may help explain the improved biological activity, although more research is needed to prove this inference.

**TABLE 6:** Quantitative results of the effect of LS-102 and AGS IV on the prevention of thrombosis in zebrafish (*n* = 10).

Groups	Concentrations (*µ*g/ml)	Staining intensity of cardiac red blood cells (pixels) (mean ± se)	Antithrombotic effect (%)
Control	−	2305 ± 32	−
Model	−	1397 ± 45	−
Aspirin	25	2036 ± 92^***^	70^***^
AGS IV	100	1446 ± 54	5
LS-102	6.25	1280 ± 119	−13
	12.5	1521 ± 72	14
	25	1597 ± 65	22
	50	1796 ± 54^***^	44^***^
	100	1950 ± 33^***^	61^***^

^***^ *p* < 0.001, vs. Model group.

## 5 Conclusion

The results of interaction studies show that LS-102 displays moderate binding of HSA by experimental and *in silico* methods, and there are no clear conformation changes, meaning that this newly synthesized drug can be considered to have no side effects. The substitution of the carboxyl group in LS-102 may be a factor that exhibited allosteric regulation on binding properties. As LS-102 showed protective effects on the heart and appropriate pharmacokinetic properties, our results are important for understanding its overall distribution, metabolism and efficacy in the human body at the molecular level. Moreover, our results highlight its safety and promising drug treatment efficacy, thus providing crucial information for clinical medicine. Ultimately, this simple and validated method provides enhanced support for drug discovery and development, while having the advantages of being less costly and time-consuming. In zebrafish experiments, LS-102 was shown to ameliorate astemizole-induced heart rate slowing and SV-BA interval increase in zebrafish and prevent arachidonic acid-induced thrombosis in zebrafish. These findings lay the foundation for further research on the mechanism of action and targets of LS-102 and provide a valuable experimental basis for future clinical applications. Moreover, our results suggest that computational simulation is consistent with experimental findings, thereby confirming the establishment of a validated and convenient method to study the molecular interaction between HSA and its ligands.

## Data Availability

The raw data supporting the conclusion of this article will be made available by the authors, without undue reservation.
