# Supplementary figures and images for: Elucidation of the binding mechanism of astragaloside IV derivative with human serum albumin and its cardiotoxicity in zebrafish embryos
Source: Front Pharmacol. 2022 Sep 23;13:987882. doi: 10.3389/fphar.2022.987882 (PMC9537572; doi:10.3389/fphar.2022.987882)

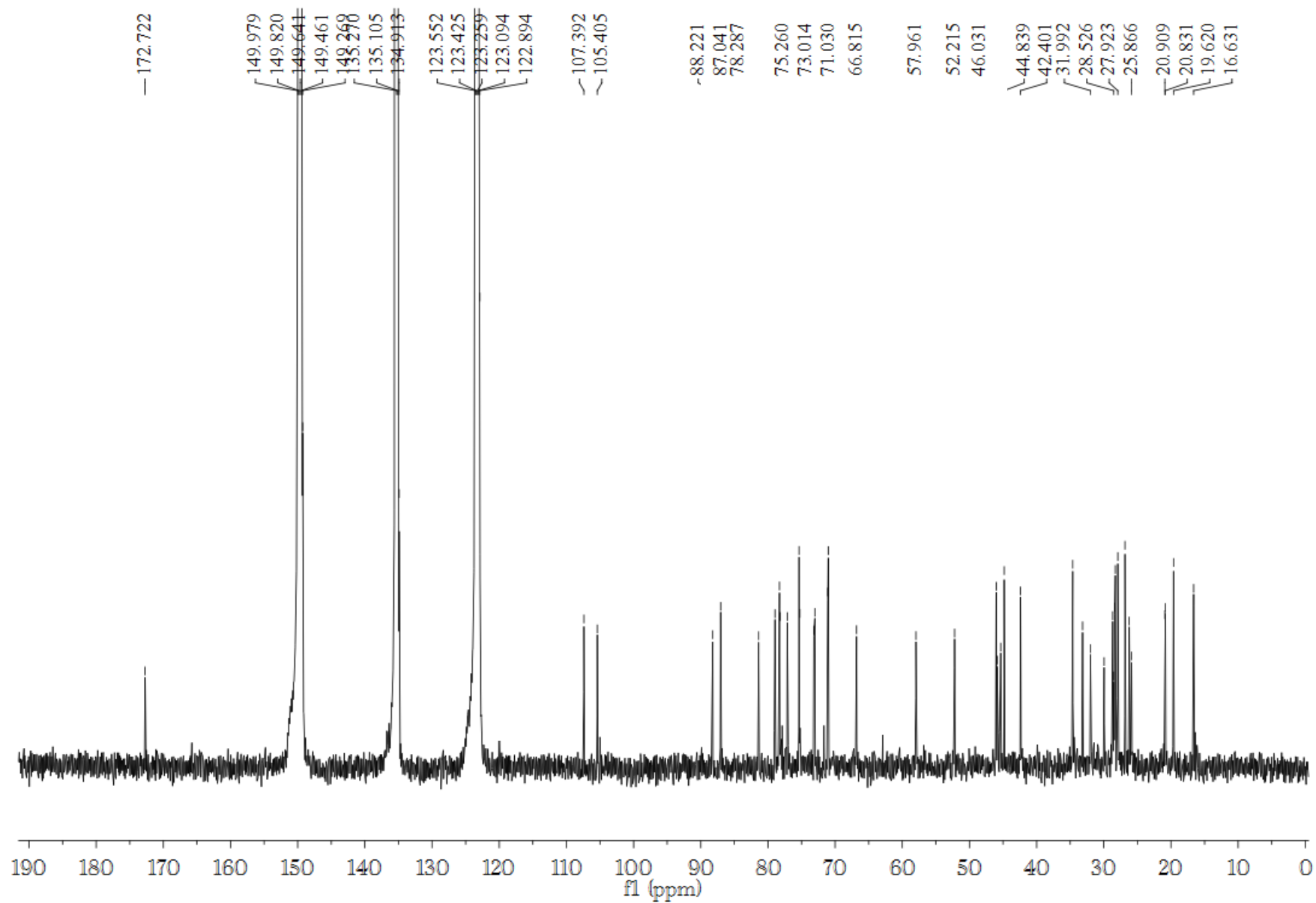

Spectrum from 798--.wiff (sample 2) - 798--, -TOF MS (100 - 2000) from 1.038 to 1.288 min

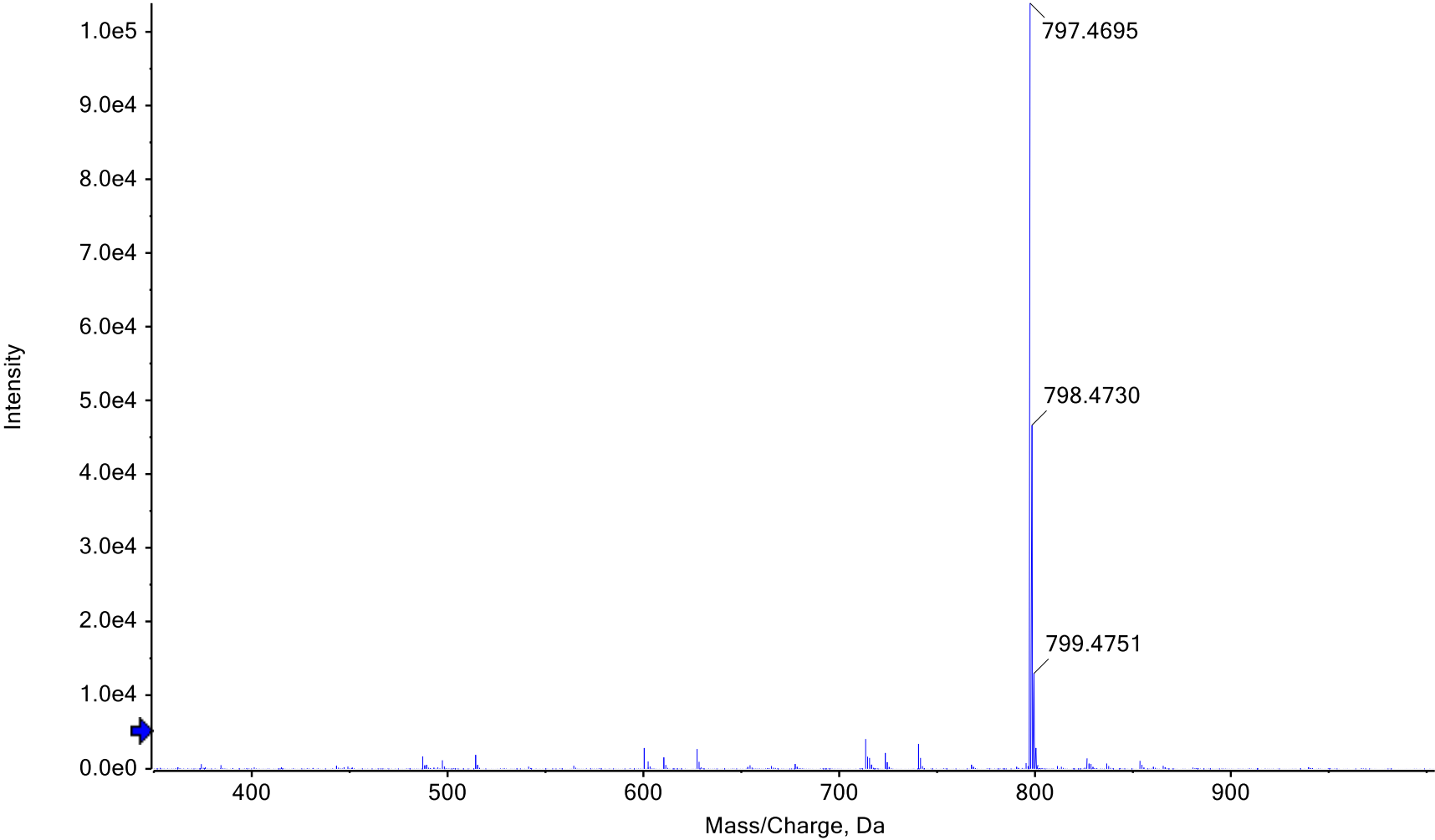

Supplement: Supplementary file 1 [file DataSheet2.pdf]
