# Supplementary material for: Elucidation of the binding mechanism of astragaloside IV derivative with human serum albumin and its cardiotoxicity in zebrafish embryos
Source: Front Pharmacol. 2022 Sep 23;13:987882. doi: 10.3389/fphar.2022.987882 (PMC9537572; doi:10.3389/fphar.2022.987882)
Supplement: Supplementary file 2 [file DataSheet1.docx]

Table S1. The regression equation of AGS IV and LS-102.

| Analyte | Sample | Regression equation | r^2^ |
| --- | --- | --- | --- |
|  |  |  | Correlation coefficient |
| AGS IV | I | Y=1.2313x+0.449 | 0.9991 |
|  | E | Y=163.27x+2.8735 | 0.9995 |
| LS-102 | I | Y=105.17x+0.8449 | 0.9991 |
|  | E | Y=1.7968x+0.0114 | 0.9995 |

I for internal dialysate; E for external dialysate.

Table S2. The accuracy, precision, and extraction recovery value for AGS IV and LS-102.

| Analyte | Sample | Concentration | Precision | Accuracy | Recovery |
| --- | --- | --- | --- | --- | --- |
|  |  | mg/mL | % | % | % |
| AGS IV | I | 0.06 | 7.15 | 10.99 | 85.08 |
|  |  | 0.10 | 8.21 | 11.21 | 117.42 |
|  |  | 0.15 | 5.25 | 5.00 | 92.17 |
|  | E | 0.06 | 3.23 | 6.42 | 97.72 |
|  |  | 0.10 | 3.60 | 5.89 | 105.91 |
|  |  | 0.15 | 2.89 | 5.74 | 92.06 |
| LS-102 | I | 0.06 | 9.23 | 11.32 | 89.21 |
|  |  | 0.10 | 11.56 | 10.74 | 100.64 |
|  |  | 0.15 | 8.22 | 13.22 | 93.21 |
|  | E | 0.06 | 8.04 | 10.28 | 94.58 |
|  |  | 0.10 | 7.55 | 10.49 | 114.67 |
|  |  | 0.15 | 7.50 | 9.79 | 97.85 |

I for internal dialysate; E for external dialysate.

Table S3. ASA for the residues of HAS, HSA-AGS IV and HSA-LS102 in Å^2^.

| Residues | ASA for HSA | ASA for HSA-AGS IV | ASA for HSA-LS102 | ΔASA for HSA-AGS IV | ΔASA for HSA-LS102 | Change of ASA (%) for HSA-AGS IV | Change of ASA (%) for HSA-LS102 |
| --- | --- | --- | --- | --- | --- | --- | --- |
| Ser-192 | 18.22 | 10.33 | 10.33 | 7.89 | 7.819 | 43.31 | 43.31 |
| Lys-195 | 72.28 | 6.05 | 6.88 | 66.24 | 65.40 | 91.64 | 90.48 |
| Gln-196 | 35.94 | 21.90 | 20.73 | 14.04 | 15.21 | 39.07 | 42.32 |
| Leu-198 | 104.98 | 4.99 | 4.99 | 99.99 | 99.99 | 95.24 | 95.24 |
| Lys-199 | 35.94 | 2.69 | 3.03 | 33.25 | 32.91 | 92.52 | 91.57 |
| Trp-214 | 104.98 | 23.17 | 24.45 | 81.81 | 80.53 | 77.93 | 76.71 |
| Arg-257 | 43.30 | 16.05 | 15.23 | 27.25 | 28.07 | 62.92 | 64.83 |
| His-288 | 43.44 | 24.02 | 24.64 | 19.42 | 18.80 | 44.71 | 43.28 |
| Ala-291 | 68.67 | 26.60 | 25.92 | 42.07 | 42.76 | 61.27 | 62.26 |
| Glu-292 | 126.67 | 69.43 | 67.11 | 57.24 | 59.56 | 45.19 | 47.02 |
| Glu-450 | 32.08 | 27.29 | 27.57 | 4.78 | 4.50 | 14.91 | 14.05 |
| Asp-451 | 55.55 | 5.29 | 5.10 | 50.26 | 50.45 | 90.49 | 90.82 |

| Table S4. The per cent values for secondary structures. | | | | | | | |
| --- | --- | --- | --- | --- | --- | --- | --- |
|  | Structure | Coil | Bend | Turn | A-Helix | 5-Helix | 3-Helix |
| AGS IV | 0.77 | 0.14 | 0.06 | 0.07 | 0.69 | 0.01 | 0.02 |
| LS-102 | 0.78 | 0.14 | 0.05 | 0.08 | 0.70 | 0.01 | 0.02 |

Table S5. MTC result of LS-102（n=30）.

| Groups | Concentrations（*µ*g/mL） | Total (tails) | Deaths (tails) | Mortality（%） |
| --- | --- | --- | --- | --- |
| Control | - | 30 | 0 | 0 |
| LS-102 | 1 | 30 | 0 | 0 |
|  | 10 | 30 | 0 | 0 |
|  | 100 | 30 | 0 | 0 |
|  | 250 | 30 | 4 | 13.3 |
|  | 500 | 30 | 30 | 100 |

Table S6. Phenotypic statistics of LS-102 and AGS IV on zebrafish after induction with astemizole（n=30）.

| Inducer concentrations | Group | Concentration（*µ*g/mL） | Mortality（%） | Phenotype |  |
| --- | --- | --- | --- | --- | --- |
|  | Control | - | 0 | No abnormalities seen |  |
| 4 *µ*M | Model | - | 0 | 100% cardiac capsular oedema, lack of blood flow |  |
|  | AGS IV | 250 | 3.3 | 100% cardiac capsular oedema, lack of blood flow |  |
|  | LS-102 | 27.8 | 0 | 100% cardiac capsular oedema, lack of blood flow |  |
|  |  | 83.3 | 0 | 83.3% pericardial oedema, venous sinus stasis; 26.7% absence of blood flow; 56.7% slowed blood flow |  |
|  |  | 250 | 30 | No abnormalities seen |  |
